# Psychometric Properties and Factor Structure of the Spanish Version of the Acceptance and Action Questionnaire-II (AAQ-II) in Ecuador

**DOI:** 10.3390/ijerph18062944

**Published:** 2021-03-13

**Authors:** Belén Paladines-Costa, Víctor López-Guerra, Pablo Ruisoto, Silvia Vaca-Gallegos, Raúl Cacho

**Affiliations:** 1 Department of Psychology, Technical Particular University of Loja (UTPL), Loja 110107, Ecuador; mbpaladines@utpl.edu.ec (B.P.-C.); vmlopez5@utpl.edu.ec (V.L.-G.); slvaca@utpl.edu.ec (S.V.-G.); 2 Department of Psychology, National University of Distance Education (UNED), 28015 Madrid, Spain; 3 Department of Health Sciences, Public University of Navarre, 31006 Pamplona, Spain; raul.cacho@unavarra.es

**Keywords:** acceptance and action questionnaire, acceptance and commitment therapy, psychological inflexibility, psychometric properties, university students

## Abstract

(1) Background: The Acceptance and Action Questionnaire-II (AAQ-II) is the most well-known self-report measure to assess psychological inflexibility, a transdiagnostic pathological process, and targets for interventions. Objective: The aim of this study was to analyze the psychometric properties and factorial structure of the Ecuadorian Spanish version of the AAQ-II in a large sample of college students in Ecuador. (2) Methods: A total of 7905 students, 46.26% male and 53.75% female, from 11 Ecuadorian universities were surveyed. The AAQ-II was tested for factorial structure, reliability, and correlations with other health-related measures. (3) Results: The AAQ-II showed an unidimensional factorial structure, accounting for 66.87% to 70% of the total variance and showing a good fit of the data to the model (comparative adjustment index (CFI) = 0.995; goodness of fit index (GFI) = 0.992; Standardized Root Mean Squared Residual (SRMR) = 0.037; mean square approximation error (RMSEA) = 0.047, CI90% = 0.038–0.056). Reliability was optimal (Cronbach’s α = 0.919; ω = 0.928), and AAQ-II scores significantly correlated with multiple health indicators. Psychological inflexibility was significantly higher in women than men. (4) Conclusions: The Spanish version of the AAQ-II showed good psychometric properties, which further supports psychological inflexibility, not just as a transdiagnostic process.

## 1. Introduction

Psychological inflexibility (PI) is characterized by a tendency to act rigidly, avoiding undesirable aversive events and producing important limitations in a person’s life [1,2,3]. PI is composed of a set of the subprocess, including experiential avoidance, in which individuals seek to avoid, escape, or otherwise control the occurrence of difficult thoughts and feelings, despite the harmful consequences of doing so [4,5]. It is transdiagnostic in nature and a core concept in the acceptance and commitment therapy (ACT) model [4], which is probably one of the most representative of the third-generation therapies and has the most empirical support [6,7].

Nowadays, the Acceptance and Action Questionnaire is the gold standard in the measurement of psychological inflexibility (PI) [1]. The first version, developed by Hayes [8], was a short scale that had between 9 and 16 items depending on the version, like a Likert-style scale. Its internal consistency was just satisfactory (α = 0.70), and the test-retest reliability was 0.64 over 4 months. In other samples (such as people with low academic levels or whose second language was English), the reliability indices were lower. The factorial structure of the Acceptance and Commitment Questionnaire (AAQ-I) was also unstable. For example, Hayes [8] reported a one-dimensional factorial structure for the 9 and 16-item versions, while Bond and Bruce [9] found a bifactorial structure for the 16-item version. Considering the inconsistencies and contradictions in the psychometric results of the first version, Bond et al. [1] proposed the second version of the Acceptance and Commitment Questionnaire (AAQ-II), which achieved better psychometric properties compared to the first version [1] and showed good internal consistency for both clinical and non-clinical samples (α = 0.78–0.88); adequate test–retest reliability at 3 and 12 months (α = 0.81 and 0.79, respectively); a one-dimensional factorial structure; and satisfactory convergent, concurrent, and discriminative validities [1].

In the last decade, a number of Acceptance and Commitment Questionnaire (AAQ-II) versions have been developed for European (Portugal [3], Spain [6], Germany [10], Hungary [11], and Greece [12]), Asian (China [13] and Malaysia [14]), Middle Eastern (Turkey [15]), and large South American (Brazil [16] and Colombia [7]) countries.

The first Spanish translation of the AAQ-I scale (9 items) was carried out by Barraca [17], which presents problems related to internal consistency and factorial structure similar to those reported in the first version of the scale in English by Hayes [8]. Therefore, most studies on PI conducted in Spanish-speaking countries have used the AAQ-II translation [6].

Previous studies have validated the AAQ-II in Spain and Colombia by Ruiz et al. [6,7], but its psychometric properties in Ecuador remain unknown, making both psychological evaluation and research under the acceptance and commitment therapy approach difficult.

The aim of this study is to analyze the psychometric properties of an Ecuadorian-Spanish version of the AAQ-II in a large sample in Ecuador.

## 2. Materials and Methods

### 2.1. Participants

College students enrolled in 11 universities in Ecuador were invited via e-mail to participate in the study, and then they completed a computerized survey within the 3-week assessment period. A total of 7905 participants met the inclusion criteria of being enrolled in at least one whole academic year and completing the entire survey (the average response rate across universities was 47.80%, ranging from 39.10% to 56.30%). The average age was 21.49 years (SD = 3.68). Among the participants, 4249 (53.75%) were female, with an average age of 21.25 (SD = 3.70), and 3656 (46.26%) were male, with an average age of 21.80 (SD = 3.75). Among the participants, 60% came from public universities, 91% were single, and 73% were full-time students.

### 2.2. Measures

Acceptance and Commitment Questionnaire–II (AAQ-II) [1]. This is the most widely used general measure of psychological inflexibility. It consists of 7 items in which participants respond in a 7-point Likert-type scale, from 1 = “never” to 7 = “always”. Scores range from 7 to 49. Higher scores indicate a tendency to act under the need to control or avoid aversive thoughts, memories, or feelings. The Spanish version presents a unifactorial structure that explains 57.33% of the total variance of the scale responses, and the internal consistency across the different samples was good (between α = 0.75 and α = 0.93). An example item is “My painful experiences and memories make it difficult for me to live a life that I would value”. The AAQ-II scores were significantly related to general psychopathology and quality of life measures [6].

Life Engagement Test (LET) [18]. This 6-item scale measures vital commitment, which is understood as the degree to which the person engages in activities that are valuable to them as opposed to experiential avoidance. The response format consists of five alternatives (1 = strongly disagree, 2 = disagree, 3 = neutral, 4 = agree, 5 = strongly agree). Scores range from 6 to 30, with a higher score indicating a greater life engagement. This scale presents a one-dimensional structure; an adequate internal consistency in different samples ranging from α = 0.72 and α = 0.87; a test–retest reliability in the different samples (0.61 to 0.76); and satisfactory convergent, discriminant, and predictive validities [18]. Medium to strong negative correlations were expected between the AAQ-II and LET.

Patient Health Questionnaire of Depression and Anxiety (PHQ-4) [19]. This questionnaire assesses depression and anxiety associated with functional impairment and disability days. It consists of 4 items and a Likert-type response format with four options (0 = never, 1 = several days, 2 = more than half the days, 3 = almost every day). Scores range from 0 to 12. A higher score indicates a greater anxiety and depression level. The scale presented a two-dimensional structure and optimum reliability (α = 0.84) [20]. An example item is “Feeling nervous, anxious or on edge”. Medium to strong positive correlations were expected between the AAQ-II and PHQ-4.

Loneliness Scale Revised-Short (UCLA-3) [21]. This consists of a brief 3-item scale evaluating the subjective feeling of loneliness, understood as the perception of less social support being available than desired. Participants respond based on their agreement with a series of statements, where 1 = “hardly ever”, 2 = “sometimes”, and 3 = “often”. Scores range from 0 to 9. Higher scores indicate a greater feeling of loneliness or lack of social support. The internal consistency was good for both men (α = 0.75) and women (α = 0.84). An example item is “How often do you feel that you lack company?”. Medium to strong positive correlations were expected between the AAQ-II and UCLA-3.

Perceived Stress Scale (PSS-14) [22,23]. This scale consists of 14 items to assess the degree to which people perceive a lack of control in their daily lives. Participants respond to a 5-point Likert-type scale ranging from 0 (“never”) to 4 (“very often”). Scores range from 0 to 56 points. Higher scores indicate higher levels of psychological stress. In the Ecuadorian version [24], the reliability was optimal with the alpha and omega coefficients (α = 0.85 and ω = 0.80), and it presented a bifactorial structure, which explains 49.46% of the total variance, and satisfactory convergent validity with multiple health indicators. An example item is “In the last month, how often have you been upset because of something that happened unexpectedly?”. Medium to strong positive correlations were expected between the AAQ-II and PSS-14.

Brief Resilience Scale (BRS) [25]. This assesses the individual’s ability to adapt and recover from stressful and adverse situations. It consists of 6 items with 5 Likert-type alternatives of responses, ranging from 1 = “strongly disagree” and 5 = “strongly agree.” This scale presents an adequate internal reliability (α = 0.83); a one-dimensional internal structure; and satisfactory concurrent, convergent, and predictive validities [26]. An example item is “I tend to bounce back quickly after hard times”. Medium to strong negative correlations were expected between the AAQ-II and BRS.

Depression and anxiety symptomatology (PHQ-4 scores), loneliness (UCLA-3), psychological stress (PSS-14), resilience (BRS), and PI (AAQ-II scores) are all well-established health-indicators. PI mediates the relation between depressogenic schemas and depressive symptoms [27], and it has been related to both anxiety and psychological stress [28]. PI has also mediated the association between emotion regulation and loneliness [29].

### 2.3. Design and Procedure

A descriptive cross-sectional study was conducted. Participation was confidential and fully anonymous. A brief summary of individual scores was freely provided after the completion of the survey to encourage honest answers and a higher response rate. The study was approved by the Ethics Committee for Research in Human Beings (*Comité de Ética de Investigación en Seres Humanos*, CEISH) of the Ministry of Public Health of the Republic of Ecuador and was conducted according to the principles expressed in the Declaration of Helsinki. Written informed consent was obtained from all participants. A language adaptation of the original AAQ II into Ecuadorian Spanish was carried out by a panel of three experts (see Appendix A). The final Ecuadorian Spanish adaptation was tested on 30 candidates as a pilot trial to test for the clarity and comprehensiveness of the questionnaire.

### 2.4. Data Analysis

The statistical analyses were carried out using the IBM Statistical Package for the Social Sciences (SPSS) software (IBM Inc., Chicago, IL, USA), AMOS version 25.0 (IBM Inc., Armonk, NY, USA), and FACTOR version 10.8.03 for Windows (Rovira I Virgili University, Tarragona, Spain).

Firstly, the factorial structure was analyzed by performing an exploratory (EFA) and confirmatory (CFA) analysis. Following the recommendations of Lloret, Ferreres, Hernández and Tomás [30], and Harrington [31] that indicate that to obtain the factorial structure, both the EFA and the CFA must be performed in different samples, the total sample was divided into two random subsamples through the statistical program SPSS 25.0 (*n* = 4691) and (*n* = 3176). The chi-square statistical test did not reveal significant differences in both subsamples, so the random selection helped maintain the same proportion of sociodemographic characteristics in each one of them. Kolmogorov–Smirnov normality and Levene’s homoscedasticity tests were also conducted for basic assumptions. Based on scores distribution, a MANOVA test was conducted, which has the advantage of reducing type I error by analyzing the differences by sex of all items in the same analysis.

For the EFA, the principal component method with factor extraction and the oblimin method for the rotation of the component matrix, retaining factor loads greater than 0.40 in the rotated matrix, were used. The two criteria to identify the number of factors of the scales were Cattell eigenvalues greater than the point of change of slope in the sedimentation curve and the number of observed eigenvalues, and the sedimentation curve for the 95th percentile in one series of randomly generated matrices (Optimized Horn Parallel Analysis).

For the CFA, the maximum likelihood method was used, and the estimators of the goodness of fit were the chi ratio square by degrees of freedom (CMIN/DF), the Bentler comparative adjustment index (CFI), the Jöreskog and Sörbom goodness of fit index (GFI), the fit index Bentler–Bonett norm (NFI), the parsimony standard adjustment index (PNFI), Akaike information criterion (AIC), and the mean square approximation error (RMSEA) and its confidence intervals (CI: 90). Good fitness of the model was considered if CMIN/DF ≤ 3; CFI, GFI, and NFI ≥ 0.95; and RMSEA ≤ 0.06.

Secondly, the AAQ-II factorial invariance analysis was performed for the total, male and female, sample: configurational invariance (M1), which indicates a factorial structure without restrictions (baseline); metric invariance (M2), where equivalence restrictions are established between factor loads; strong invariance (M3) or load and intercept equivalence restrictions; and strict invariance (M4), taking into account the equivalence restrictions of factor loads, intercepts, and residuals. The measurement invariance and its levels were evaluated according to the recommendations of Cheung and Rensvold [32]: ΔCFI ≤ 0.01 and ΔRMSEA ≤ 0.015.

Thirdly, internal consistency was analyzed based on the calculation of Cronbach’s α and McDonald’s ω, considering values of 0.70 acceptable for both cases.

Finally, convergent and divergent validities were analyzed based on Pearson’s correlation between AAQ-II score and scores on scales corresponding to different well-established psychological health indicators.

## 3. Results

### 3.1. Total and Sex Differences in Acceptance and Action Questionnaire-II Scores (AAQ-II) in Ecuador

Female participants reported significantly higher levels of psychological inflexibility than males on the total score and for each individual item (Table 1).

### 3.2. Exploratory Factorial Analysis

Kaiser–Meyer–Olkin (KMO, 0.897) and Bartlett’s sphericity tests were significant (χ^2^ = 21,535.931; *p* < 0.001) [21], indicating the interrelation of the data and the relevance of continuing with the EFA. From the set of items, a single factor with an eigenvalue greater than 1 (Eigenvalue = 4.681) was obtained, which explains 66.87 of the total variance of the responses to the instrument. The factorial loads ranged between a value of 0.786 (item 2) and 0.847 (item 4) (Table 2).

The optimized Horn’s parallel analysis (PA) revealed a unifactorial structure, which explained 70.03% of the total variance. The unidimensional structure was supported (UniCo = 0.986; (CI 95%: 0.983–0.989); efficient common variance (ECV) = 0.908; (CI 95%: 0.898–0.916) and Mean of Item Residual Absolute Loadings (MIREAL) = 0.215; (CI 95%: 0.208–0.226)).

### 3.3. Confirmatory Factorial Analysis

**Figure 1:**
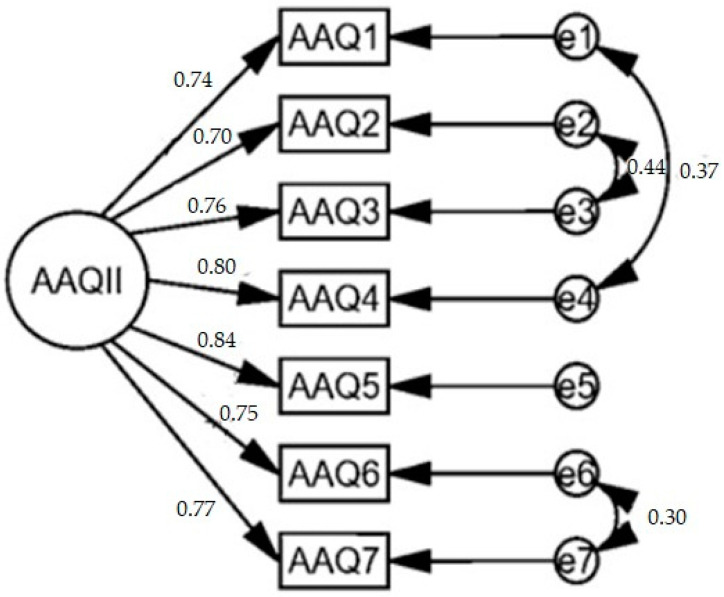
AAQ-II one-factor model.

The initial CFA showed poor fit of the data to the model (CMIN/DF = 78.236; CFI = 0.906; GFI= 0.895; NFI = 0.905; PNFI = 0.453; AIC = 1137.298; RMSEA = 0.156 (CI 90%: 0.148–0.164)). However, three correlated errors were reported between items 1 and 4, 2 and 3, and 6 and 7. Considering these correlated errors, a second model was evaluated, reporting an adequate fit of the data (CMIN/DF = 8.787. CFI = 0.993; GFI = 0.993; NFI = 0.992; PNFI = 0.390; AIC = 144.65; RMSEA = 0.050 (CI 90%: 0.041–0.059)). The standardized factorial loads of this solution ranged between 0.704 and 0.844 while being significant (*p* < 0.05), with an average of 0.766, which is higher than the required value of 0.70 [8]. The intra-item correlations do not exceed the value of 0.90 (there is no multicollinearity), and the average variance extracted (AVE) value was acceptable (0.58) (Figure 1).

The adjustment indices of the unifactorial model with correlated errors, both for the total sample and separated by sex, are presented in Table 3, showing an adequate fit in each of them. Next, the configurational invariance (M1) was analyzed, presenting good fit indicators (χ^2^ (22) = 226.240; CFI = 0.994 and RMSEA = 0.034 (0.030–0.038)). The metric invariance (M2) resulted in good fit indices (CFI = 0.994; RMSEA = 0.031), being similar to the M1 values because they presented minimal differences (ΔCFI = 0.000 and ΔRMSEA = 0.003). These results indicate that the factorial loads are invariant between the groups of men and women, and, therefore, the covariances can be compared. The strong invariance (M3) shows indices equal to the previous model (CFI = 0.994; RMSEA = 0.030) without minimal differences (ΔCFI = 0.000 and ΔRMSEA = 0.001), accepting the invariance between the thresholds. Finally, the strict invariance (M4) reflects a good fit (CFI = 0.993; RMSEA = 0.029) with minimal differences (ΔCFI = 0.001 and ΔRMSEA = 0.001), verifying the invariance of the residuals. The combined results indicate factorial invariance of the AAQ-II across both genders.

### 3.4. Internal Consistency

The internal consistency of the instrument for the one-dimensional model in Cronbach’s alpha (α = 0.919 (CI 95% = 0.916–0.921)) and omega (ω = 0.928) coefficients were adequate.

### 3.5. Convergent and Divergent Validities

The results presented in Table 4 show positive relationships between PI and perceived stress, loneliness, presence of anxiety and/or depression symptoms (convergent validity), and negative associations with resilience and life engagement (divergent validity), all being statistically significant (*p* < 0.01). The effect size of PI on the health indicators was moderate or large (r > 0.50).

## 4. Discussion

This is the first study to examine the psychometric properties of the golden self-report measure of psychological inflexibility in an adult Ecuadorian sample. Furthermore, to our knowledge, this is the largest sample assessed with this questionnaire in Latin America. Overall, the results showed adequate psychometric properties, supporting its use in Ecuador.

The Ecuadorian Spanish version of the AAQ-II scale has shown a one-dimensional factorial structure consistent with the original version, high internal consistency, and good convergent and divergent validities. Female participants reported significantly higher psychological inflexibility than males. These results are consistent with those previously reported by Ruisoto et al. [33], Ruiz et al. [7], and Martins and Giardini [16].

Regarding factorial structure, both EFA and PA indicated that the one-dimensional structure accounts for between 66.87% and 70.03% of the total variance. Other indicators, such as UniCo, ECV, and MIREAL, proposed by Ferrando and Lorenzo-Seva [34], also suggested unidimensionality, consistent with the original study [1] and with other versions (Hungary [11], Greece [12], Brazil [16], Spain [6], and Colombia [7]). Moreover, the CFA also reflected a good fit of the data to the one-dimensional structure, consistent with the results of the original study [1]. Interestingly, this study found correlated errors between items 1 and 4, 2 and 3, and 6 and 7.

Previous studies had only detected correlated errors between items 1 and 4 [7,11,12,13,35], 2 and 3 [11,12], and 2 and 5 [1] but failed to detect errors between items 6 and 7. The incorporation of these correlated errors resulted in a model with a better fit to the data than that originally proposed by Bond et al. [1] and Ruiz et al. [7]. The factorial structure remained invariant in men and women [36]. Gender differences in AAQII scores suggest differences in psychological inflexibility instead of a bias in the measurement itself [37].

The internal consistency was extremely high, with values of α = 0.919 and ω = 0.928, similar or even higher than those reported in the analysis of the internal consistency or reliability of other AAQII versions [7,11,12,13,16,17,18,19,20,21,22,23,24,25,26,27,28,29,30,31,32,33,34,35,36,37,38].

The convergent and divergent validities of the Ecuadorian Spanish AAQ II were also consistent with previous studies, in which AAQ-II scores were positively correlated with the presence of more symptoms of depression [7,12,19,38,39] and anxiety [7,11,16,40], greater psychological stress [11,24,41], and greater loneliness [42], and negatively with resilience [16]. This evidence is important because it empirically supports the hypothesis of psychological inflexibility as a transdiagnostic dimension in the field of mental health.

These results should be considered with caution since the sample corresponds to university students. Future studies should further explore the psychometric properties of the Ecuadorian Spanish version of AAQII in clinical populations and other age ranges.

## 5. Conclusions

The Spanish version of the AAQ-II presents a one-dimensional structure, accounting for 67 to 70% of the variance, with good reliability and convergent and divergent validities. The scale is invariant by sex, and, thus, it can be used with guarantees in men and women. These results support the use of this scale to measure psychological inflexibility in Ecuador.

Finally, differences in the AAQ II scores reported between the Spanish and Ecuadorian versions might either shed some light on relevant cultural differences about how to cope or manage adverse private events or how this core transdiagnostic process has changed over time. However, although both implications are clinically relevant from a mental health perspective, further studies are needed to explore this path in depth.

## Figures and Tables

**Table 1:** Sex differences in AAQ-II scores.

Items	Total Sample (*n* = 7905)	Female (*n* = 4249)	Male (*n* = 3656)	F	*p*
	M (±SD)	M (±SD)	M (±SD)		
1. My painful experiences and memories make it difficult for me to live a life that I would value.	2.94 (±1.00)	3.08 (±1.65)	2.77 (±1.60)	67.726	<0.001
2. I am afraid of my feelings	3.37 (±1.84)	3.58 (±1.86)	3.13 (±1.79)	118.583	<0.001
3. I worry about not being able to control my worries and feelings.	3.45 (±1.79)	3.69 (±1.79)	3.18 (±1.75)	164.484	<0.001
4. My painful memories prevent me from having a fulfilling life	2.72 (±1.79)	2.89 (±1.85)	2.51 (±1.71)	89.096	<0.001
5. Emotions cause problems in my life.	3.22 (±1.79)	3.39 (±1.80)	3.02 (±1.75)	80.973	<0.001
6. It seems like most people are handling their lives better than I am.	3.18 (±1.88)	3.22 (±1.90)	3.13 (±1.75)	4.980	<0.026
7. Worries get in the way of my success	3.09 (±1.76)	3.18 (±1.77)	2.99 (±1.75)	19.781	<0.001
Total score	21.98 (±10.3)	23.0 (±10.4)	20.8 (±10.0)	97.274	<0.001

**Table 2:** Exploratory factorial analysis of the Acceptance and Action Questionnaire (AAQ)-II Scale.

Acceptance and Action Questionnaire (AAQ)-II Scale	(Factorial Loads)
*Item 1.* My painful experiences and memories make it difficult for me to live a life that I would value.	0.806
*Item 2.* I am afraid of my feelings.	0.786
*Item 3.* I worry about not being able to control my worries and feelings.	0.826
*Item 4.* My painful memories prevent me from having a fulfilling life.	0.847
*Item 5.* Emotions cause problems in my life.	0.845
*Item 6.* It seems like most people are handling their lives better than I am.	0.793
*Item 7.* Worries get in the way of my success.	0.820
Total explained variance: 66.866%. Eigenvalue (>1): 4.681. Extraction method: principal component analysis.	
Keiser–Meyer–Olkin (KMO) measure of sampling fitness: 0.897. Bartlett’s sphericity test (approximate chi-square): 21,535.931. Degrees of freedom: 21. Significance level: *p* < 0.001.	

**Table 3:** AAQ-II factorial invariance for the total sample and by sex.

Model	*χ* ^2^	df	C-M	Δχ^2^	Δ*df*	CFI	ΔCFI	SRMR	RMSEA (CI 90%)	ΔRMSEA
Entire Group	205.780	11	-	-	-	0.995	-	0.037	0.047 (0.042, 0.053)	-
Men	90.775	11	-	-	-	0.995	-	0.031	0.045 (0.036, 0.053)	-
Women	135.465	11	-	-	-	0.994	-	0.046	0.052 (0.04, 0.060)	-
M1	226.240	22	-	-	-	0.994	-	0.039	0.034 (0.030, 0.038)	-
M2	236.939	28	M2–M1	10.699	6	0.994	0	0.050	0.031 (0.027, 0.034)	0.003
M3	241.625	29	M3–M2	4.868	1	0.994	0	0.080	0.030 (0.027, 0.034)	0.001
M4	297.692	39	M4–M3	56.067	10	0.993	0.001	0.097	0.029 (0.026, 0.032)	0.001

Note: comparison of factorial invariance models (C-M); comparative adjustment index (CFI); Standarized Root Mean-Square *(SRMR);* mean square approximation error (RMSEA); Δ = increase; confidence intervals (CI).

**Table 4:** Correlation matrix between AAQ-II scores and other psychological health-related measures.

Measure	Perceived Stress	Loneliness	Anxiety and Depression	Life Engagement	Resilience
AAQII	0.676 *	0.640 *	0.592 *	−0.480 *	−0.574 *

Note: Psychological inflexibility (AAQII). * *p* < 0.01.

## Data Availability

The data presented in this study are available on request from the corresponding author. The data are not publicly available due to their containing information that could compromise the privacy of research participants.

## Appendix A

AAQ-II adapted Spanish version from Ruiz et al. [6]
*1.* Mis experiencias y recuerdos dolorosos hacen que me sea difícil vivir la vida que querría
*2.* Tengo miedo de mis sentimientos
*3.* Me preocupa no ser capaz de controlar mis preocupaciones y sentimientos
*4.* Mis recuerdos dolorosos me impiden llevar una vida plena
*5.* Mis emociones interfieren en cómo me gustaría que fuera mi vida
*6.* Parece que la mayoría de la gente lleva su vida mejor que yo
*7.* Mis preocupaciones interfieren en el camino de lo que quiero conseguir

## References

[1] Bond F.W., Hayes S.C., Baer R.A., Carpenter K.M., Guenole N., Orcutt H.K., Waltz T., Zettle R.D. (2011). Preliminary psychometric properties of the Acceptance and Action Questionnaire–II: A revised measure of psychological inflexibility and experiential avoidance. Behav. Ther..

[2] López M. (2014). Estado actual de la terapia de aceptación y compromiso en adicciones. Health Addict..

[3] Costa J., Marôco J., Pinto-Gouveia J., Galhardo A. (2014). Validation of the psychometric properties of Acceptance and Action Questionnaire-II in clinical and nonclinical groups of Portuguese population. Int. J. Psychol. Psychol. Ther..

[4] Hayes S.C., Wilson K.G., Gifford E.V., Follette V.M., Strosahl K. (1996). Experiential avoidance and behavioral disorders: A functional dimensional approach to diagnosis and treatment. J. Consult. Clin. Psychol..

[5] Hayes S.C. (2004). Acceptance and commitment therapy, relational frame theory, and the third wave of behavioral and cognitive therapies. Behav. Ther..

[6] Ruiz F.J., Langer A.I., Luciano C., Cangas A.J., Beltrán I. (2013). Measuring experiential avoidance and psychological inflexibility: The Spanish translation of the Acceptance and Action Questionnaire. Psicothema.

[7] Ruiz F.J., Suárez-Falcón J.C., Cárdenas-Sierra S., Durán Y., Guerrero K., Riaño-Hernández D. (2016). Psychometric properties of the Acceptance and Action Questionnaire-II in Colombia. Psychol. Rec..

[8] Hayes S.C., Strosahl K., Wilson K.G., Bissett R.T., Pistorello J., Toarmino D., Polusny M.A., Dykstra T.A., Batten S.V., Bergan J.R. (2004). Measuring experiential avoidance: A preliminary test of a working model. Psychol. Rec..

[9] Bond F.W., Bunce D. (2000). Mediators of change in emotion-focused and problem-focused worksite stress management interventions. J. Occup. Health Psychol..

[10] Gloster A.T., Klotsche J., Chaker S., Hummel K.V., Hoyer J. (2011). Assessing psychological flexibility: What does it add above and beyond existing constructs?. Psychol. Assess..

[11] Eisenbeck N., Szabó-Bartha A. (2018). Validation of the Hungarian version of the Acceptance and Action Questionnaire-II (AAQ-II). J. Context. Behav. Sci..

[12] Karekla M., Michaelides M. (2017). Validation and invariance testing of the Greek adaptation of the Acceptance and Action Questionnaire -II across clinical vs. nonclinical samples and sexes. J. Context. Behav. Sci..

[13] Zhang C., Chung P., Si G., Liu J. (2014). Psychometric properties of the Acceptance and Action Questionnaire–II for Chinese college students and elite Chinese athletes. Meas. Eval. Couns. Dev..

[14] Shari N., Zainal N., Guan N., Ahmad Z., Yahaya N. (2019). Psychometric properties of the acceptance and action questionnaire (AAQII) Malay version in cancer patients. PLoS ONE.

[15] Meunier B., Atmaca S., Ayrancı E., Gökdemir B.P., Uyar T., Baştuğ G. (2014). Psychometric properties of the Turkish version of the Acceptance and Action Questionnaire-II (AAQ-II). J. Evid. Based Psychother..

[16] Martins L., Giasdini S. (2015). Propriedades psicométricas iniciais do Acceptance and Action Questionnaire—II—Versão brasileira. Psico USF.

[17] Barraca J. (2004). Spanish adaptation of the Acceptance and Action Questionnaire (AAQ). Int. J. Psychol. Psychol. Ther..

[18] Scheier M.F., Wrosch C., Baum A., Cohen S., Martire L.M., Matthews K.A., Schulz R., Zdaniuk B. (2006). The Life Engagement Test: Assessing Purpose in Life. J. Behav. Med..

[19] Löwe B., Wahl I., Rose M., Spitzer C., Glaesmer H., Wingenfeld K., Schneider A., Brähler E. (2010). 4-item measure of depression and anxiety: Validation and standardization of the Patient Health Questionnaire-4 (PHQ-4) in the general population. J. Affect. Disord..

[20] Kocalevent R., Finck C., Jimenez W., Sautier L., Hinz A. (2014). Standardization of the Colombian version of the PHQ-4 in the general population. BMC Psychiatry.

[21] Hughes M.E., Waite L.J., Hawkley L.C., Cacioppo J.T. (2004). A Short Scale for Measuring Loneliness in Large Surveys. Res. Aging.

[22] Cohen S., Kamarck T., Mermelstein R. (1983). A global measure of perceived stress. J. Health Soc. Behav..

[23] Remor E. (2006). Psychometric properties of a European Spanish version of the Perceived Stress Scale (PSS). Span. J. Psychol..

[24] Ruisoto P., López-Guerra V.M., Paladines M.B., Vaca S.L., Cacho R. (2020). Psychometric properties of the three versions of the Perceived Stress Scale in Ecuador. Physiol. Behav..

[25] Smith W.B., Dalen J., Wiggins K., Tooley E., Christopher P., Bernard J. (2008). The Brief Resilience Scale: Assessing the ability to bounce back. Int. J. Behav. Med..

[26] Rodriguez R., Alonso-Tapia J., Hernansaiz H. (2016). Reliability and Validity of the Brief Resilience Scale (BRS) Spanish Version. Psychol. Assess..

[27] Ruiz F.J., Odriozola-González P. (2016). The role of psychological inflexibility in Beck’s cognitive model of depression in a sample of undergraduates. Ann. Psychol..

[28] Tavakoli N., Broyles A., Reid E.K., Sandoval J.R., Correa-Fernández V. (2019). Psychological inflexibility as it relates to stress, worry, generalized anxiety, and somatization in an ethnically diverse sample of college students. J. Context. Behav. Sci..

[29] Smith B.M., Twohy A.J., Smith G.S. (2020). Psychological inflexibility and intolerance of uncertainty moderate the relationship between social isolation and mental health outcomes during COVID-19. J. Context. Behav. Sci..

[30] Lloret-Segura S., Ferreres-Traver A., Hernandez-Baeza A., Tomas-Marco I. (2014). Exploratory item factor analysis: A practical guide revised and updated. An. Psicol..

[31] Harrington D. (2009). Confirmatory Factor Analysis.

[32] Cheung G.W., Rensvold R.B. (2002). Evaluating Goodness-of-Fit Indexes for Testing Measurement Invariance. Model. Struct. Equ..

[33] Ruisoto P., Vaca S.L., López-Goñi J.J., Cacho R., Fernández-Suárez I. (2017). Gender Differences in Problematic Alcohol Consumption in University Professors. Int. J. Environ. Res. Public.

[34] Ferrando P.J., Lorenzo-Seva U. (2017). Assessing the Quality and Appropriateness of Factor Solutions and Factor Score Estimates in Exploratory Item Factor Analysis. Educ. Psychol. Meas..

[35] Renshaw T.L. (2018). Probing the relative psychometric validity of three measures of psychological inflexibility. J. Context. Behav. Sci..

[36] Brown T.A. (2006). Confirmatory Factor Analysis for Applied Research.

[37] Caycho-Rodríguez T., Ventura-León J., Barboza-Palomino M., Reyes-Bossio M., Gallegos W.L.A., Cadena C.H.G., Cabrera-Orosco I., Ayala J., Morgado-Gallardo K., Cahua J.C.H. (2018). Validez e invarianza factorial por sexo de una medida breve de Satisfacción con la Vida Familiar en escolares de Lima (Perú). Univ. Psychol..

[38] Vilardaga R., Estévez A., Levin M.E., Hayes S.C. (2012). Deictic relational responding, empathy, and experiential avoidance as predictors of social anhedonia: Further contributions from relational frame theory. Psychol. Rec..

[39] Kato T. (2016). Impact of psychological inflexibility on depressive symptoms and sleep difficulty in a Japanese sample. SpringerPlus.

[40] Valencia P., Paz J., Paredes E., León M., Zuñe C., Falcón C., Portal R., Cáceres R., Murillo L. (2017). Evitación experiencial, afrontamiento y ansiedad en estudiantes de una universidad pública de Lima Metropolitana. Interacciones.

[41] Wersebe H., Lieb R., Meyer A.H., Hofer P., Gloster A.T. (2018). The link between stress, well-being, and psychological flexibility during an Acceptance and Commitment Therapy self-help intervention. Int. J. Clin. Health Psychol..

[42] Shi R., Zhang S., Zhang Q., Fu S., Wang Z. (2016). Experiential Avoidance Mediates the Association between Emotion Regulation Abilities and Loneliness. PLoS ONE.

